# The regulatory effect of miRNAs is a heritable genetic trait in humans

**DOI:** 10.1186/1471-2164-13-383

**Published:** 2012-08-10

**Authors:** Paul Geeleher, Stephanie R Huang, Eric R Gamazon, Aaron Golden, Cathal Seoighe

**Affiliations:** 1 Department of Mathematics, Statistics and Applied Mathematics, National University of Ireland, Galway, Ireland; 2 Section of Hematology/Oncology, Department of Medicine, University of Chicago, Chicago, USA; 3 Section of Genetic Medicine, Department of Medicine, University of Chicago, Chicago, USA; 4 Department of Genetics, Albert Einstein College of Medicine, 1300 Morris Park Ave, Bronx, NY 10461, USA; 5 Institute of Infectious Disease and Molecular Medicine, University of Cape Town, Cape Town 7925, South Africa

## Abstract

**Background:**

microRNAs (miRNAs) have been shown to regulate the expression of a large number of genes and play key roles in many biological processes. Several previous studies have quantified the inhibitory effect of a miRNA indirectly by considering the expression levels of genes that are predicted to be targeted by the miRNA and this approach has been shown to be robust to the choice of prediction algorithm. Given a gene expression dataset, Cheng *et al.* defined the regulatory effect score (RE-score) of a miRNA as the difference in the gene expression rank of targets of the miRNA compared to non-targeted genes.

**Results:**

Using microarray data from parent-offspring trios from the International HapMap project, we show that the RE-score of most miRNAs is correlated between parents and offspring and, thus, inter-individual variation in RE-score has a genetic component in humans. Indeed, the mean RE-score across miRNAs is correlated between parents and offspring, suggesting genetic differences in the overall efficiency of the miRNA biogenesis pathway between individuals. To explore the genetics of this quantitative trait further, we carried out a genome-wide association study of the mean RE-score separately in two HapMap populations (CEU and YRI). No genome-wide significant associations were discovered; however, a SNP rs17409624, in an intron of *DROSHA*, was significantly associated with mean RE-score in the CEU population following permutation-based control for multiple testing based on all SNPs mapped to the canonical miRNA biogenesis pathway; of 244 individual miRNA RE-scores assessed in the CEU, 214 were associated (*p* < 0.05) with rs17409624. The SNP was also nominally significantly associated (*p* = 0.04) with mean RE-score in the YRI population. Interestingly, the same SNP was associated with 17 (8.5% of all expressed) miRNA expression levels in the CEU. We also show here that the expression of the targets of most miRNAs is more highly correlated with global changes in miRNA regulatory effect than with the expression of the miRNA itself.

**Conclusions:**

We present evidence that miRNA regulatory effect is a heritable trait in humans and that a polymorphism of the *DROSHA* gene contributes to the observed inter-individual differences.

## Background

microRNAs (miRNAs) are a class of small non-coding RNA molecules of approximately 21 nucleotides in length that regulate gene expression. They typically bind to complementary loci in the 3′ untranslated region (UTR) of mRNA and prevent translation to mature protein. An individual miRNA can regulate the expression of hundreds of genes. Some genes, particularly those with longer 3′ UTRs, are often the targets of multiple miRNAs and consequently, miRNA mediated regulation tends to result in the fine tuning of the expression of many proteins within a cell [1,2]. In mammals, miRNAs are thought to regulate the expression of as many as 50% of protein coding genes [3]. miRNA expression impacts on almost every cellular process and miRNA dysregulation has been implicated in many pathologies [1,4].

miRNAs regulate a range of biological pathways associated with cancer including apoptosis [5] and cell proliferation [6]; dysregulation of miRNAs has also been widely observed in cancer [7]. For example over expression of miR-155 has been implicated in Hodgkin’s and Burkitt’s lymphoma [8], while miR-15 and miR-16, which target the anti-apoptotic gene BCL2, have been shown to be dysregulated in chronic lymphocytic leukemia [9]. miRNAs have been found in many of the genomic regions associated with chromosomal abnormalities in cancer, including regions of amplification, which may contain oncogenes, regions of loss of heterozygosity, which may harbor tumor suppressor genes and fragile sites which are preferential sites for translocation, deletion, amplification, sister chromatid exchange and insertion of tumor associated viruses like human papilloma virus [10].

While many specific maturation steps have been uncovered for different miRNAs, most known human miRNAs are processed in the same way by the miRNA biogenesis pathway. miRNA precursors, known as primary miRNA (pri-miRNA) are transcribed by RNA polymerase II or III. These transcripts are subsequently cleaved by the microprocessor complex DROSHA-DGCR8 to form the pre-miRNA, which is transported from the nucleus to the cytoplasm by XPO5-RAN-GTP. There, it is cleaved by DICER1-TRBP to form the two stranded miRNA duplex; the passenger strand is detached and normally degraded, although in some cases it acts as a separate functional miRNA. The remaining functional strand combines with E1F2C2 proteins and forms the RNA-induced silencing complex (RISC). The miRNA then guides RISC to prevent translation of target mRNAs. Translation is prevented by mRNA deadenylation, mRNA target cleavage or translational repression [11]. Of the mechanisms of post-transcriptional regulation by miRNAs, lowered mRNA levels (mRNA cleavage or deadenylation) accounts for most (> 84%) of decreased protein production [12]. This implies that it is possible to assess levels of miRNA mediated gene silencing from the mRNA levels of a miRNA’s target transcripts.

Cheng *et al.* quantified miRNA activity in this way by defining the regulatory effect score (RE-score) of a miRNA in a sample as the average expression rank of genes that are not predicted to be targeted by the miRNA minus the average expression rank of the predicted targets of the miRNA [13]. Thus, the RE-score is intended to measure the extent to which targets of the miRNA are downregulated in a sample relative to other genes. It is not informative to compare the RE-scores of different miRNAs, but comparison of the RE-score of a given miRNA between samples can provide an indication of a difference in the repressive effect of the miRNA in the samples. For example, if the targets of a given miRNA relative to non-targets are ranked higher in a set of cancer samples than in comparable normal tissues, this suggests that the miRNA exerts less control over gene expression in the cancer samples. There have been numerous other studies published that have also investigated miRNA regulation by assessing changes in expression of mRNA targets [14-18].

We sought to investigate whether there is evidence of natural variation in this phenotype between human individuals using RE-scores calculated from microarray and RNA-seq data generated from the CEU (Utah residents with ancestry from northern and western Europe) and YRI (Yoruba in Ibadan, Nigeria) lymphoblastoid cell lines of the HapMap project [19-23]. Microarray data were available for 56 trios of related individuals in these populations (consisting of two parents and an offspring). We used these data to investigate the genetic component of the variation in RE-scores. Positive correlation between the value of a phenotype in an offspring and the mean value in parents provides evidence of a heritable component in the variation of the phenotype and the slope of the linear regression line relating parent mean to offspring values can be used as an estimate of the narrow-sense heritability [24-26].

## Results and discussion

### Heritability of the regulatory effect of miRNAs

Microarray data [23] were obtained for 56 trios (both parents and an offspring) from the CEU and YRI populations of the HapMap project [19,20]. Using miRNA targets predicted by TargetScan [2,27] we compared RE-scores between parents and offspring. For 51% of miRNAs the mean RE-score of parents and the RE-score of the offspring were significantly (*p* < 0.05) positively correlated (Table 1). Population of origin was included in these regressions to model biological and technical differences between the CEU and YRI cell lines. Regression p-values and slopes for heritability of individual miRNA RE-scores from TargetScan and a second miRNA prediction algorithm (PicTar [28]) are provided as Additional file 1; histograms of these p-values are shown in Additional file 2: Figure S1.

**Table 1:** Summary of results for individual miRNA RE-scores calculated using TargetScan

Number of miRNAs	244
Average number of target genes per miRNA	437
RE-score positively correlated between	235
mean of parent and offspring	
Positively correlated (*p* < 0.05)	124
Average Heritability (S.D)	0.30 (0.15)

**Figure 1:**
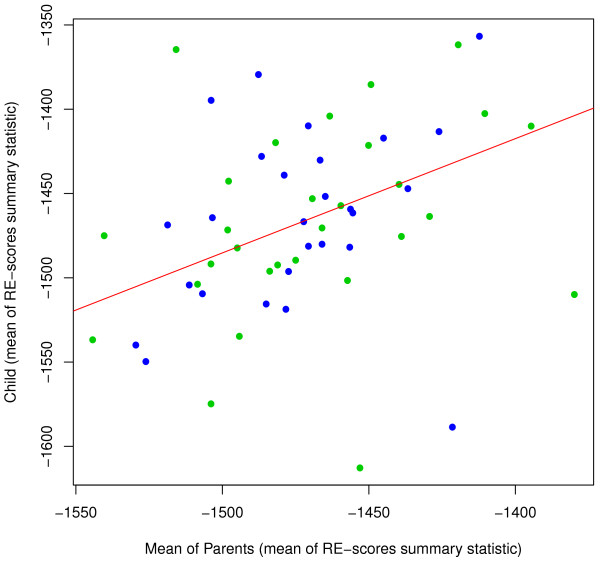
**Heritability of mean RE-score.** Scatter plots of child values of mean RE-score against mean value of parents. Points from the CEU are colored blue and YRI are green. The estimated regression line is shown in red. RE-scores were calculated using TargetScan.

We calculated the mean of the RE-score over all miRNAs. Unsurprisingly, the mean RE-score is also strongly correlated between parents and offspring in HapMap trios (Figure 1). This correlation is statistically significant using mean RE-scores calculated from targets predicted by TargetScan (*slope* = 0.68 ± 0.34; *p* = 2 × 10^−4^). The slopes of these regression lines provide estimates of the narrow-sense heritability of the mean RE-score. We also assessed mean RE-score heritability based on targets predicted by four other algorithms (which have been found to be less accurate predictors of protein levels [29]). Of these PicTar (*slope* = 0.62 ± 0.36; *p* = 1.3 × 10^−3^), miRanda [30](*slope* = 0.40 ± 0.37; *p* = 3.6 × 10^−2^) and mirTarget2 [31] (*slope* = 0.35 ± 0.32; *p* = 2.8 × 10^−2^) showed significant evidence of heritability, while one miRNA target prediction algorithm, mirBase [32], did not reach statistical significance (*slope* = 0.20 ± 0.33; *p* = 0.21).

It is possible that the apparent genetic contribution to the regulatory effect of miRNAs is a consequence of the heritability of gene expression, rather than a novel molecular phenotype. Since the expression levels of a large proportion of human genes have a strong genetic component [33-35], the correlation in RE-score between parents and offspring could simply reflect the correlation in the expression levels of a proportion of the genes targeted by the miRNA. We devised a permutation test to evaluate this possibility. For each set of mRNAs predicted to be targeted by a given miRNA we replaced predicted target genes by genes chosen at random (details in Methods). If the apparent heritability of RE-scores is merely a consequence of heritability of individual gene expression levels, the RE-scores obtained from sets of random genes should exhibit similar levels of heritability to the RE-scores based on the true predicted target sets. Greater evidence of heritability from true predicted targets compared to sets of randomly selected genes suggests that the RE-score heritability cannot be explained by the heritability of individual gene expression levels. Of 1,000 randomizations, just eight (*p* = 0.008) reached a regression p-value as extreme as the target sets predicted by TargetScan.

### Genome-wide association of mean RE-score

In order to explore the genetic contribution to RE-score variation further, we carried out a genome-wide association (GWA) test, treating mean RE-score, calculated using miRNA targets predicted by TargetScan, as a quantitative trait, and using genotype data from the HapMap project [19,20]. To avoid artifacts resulting from population structure, we carried out these tests separately on the CEU and YRI samples and excluded related individuals (offspring of the HapMap trios). RE-scores were recalculated using expression data derived from RNA-seq [21,22], which was available for parents but not for offspring of HapMap trios. Histograms and Manhattan plots of p-values are shown in Additional file 2: Figure S2. The p-value distributions show a peak towards low p-values, suggesting the presence of some true positive associations. The top ten most significantly associated loci in both populations are shown in Additional file 3. None of these associations remained significant following a permutation-based correction for multiple testing. This is not surprising given the relatively small number of samples compared to typical GWA studies.

### Association of mean RE-score with SNPs in the miRNA biogenesis pathway

Cheng *et al.*[13] used the RE-score metric to compare miRNA repression in Estrogen Receptor Positive (ER+) and Estrogen Receptor Negative (ER-) breast cancers and found that miRNAs tended to have higher RE-scores (i.e. their targets were more repressed) in the latter. The differences in RE-scores between the two cancer subtypes was attributed to dysregulation of key genes in the microRNA biogenesis pathway [13]. We used linear regression to investigate the relationships between seven key genes in the miRNA biogenesis pathway, (*DICER1*, *EIF2C2*, *DROSHA*, *DGCR8*, *XPO5*, *RAN* and *TRBP*) and mean RE-score, first using all samples from both populations pooled (including population of origin as a factor in the model) and then in each of the populations separately. Expression levels of five of these seven genes were significantly correlated with mean RE-score (Table 2), consistent with a contribution of differential regulation of the miRNA biogenesis pathway to differences in mean RE-score. In fact, a large proportion (37.8%) of all genes were significantly associated (*p* < 0.05) with mean RE-score; however, this proportion was somewhat higher (five out of seven, or 71.4%) for genes in the miRNA biogenesis pathway. Given this relationship between RE-score and the activities of genes in the miRNA biogenesis pathway these genes are worthy of closer examination for genetic association with mean RE-score.

**Table 2:** Associations between expression levels of key miRNA biogenesis genes and mean RE-score

	**CEU**		**YRI**		**Pooled**	
	**Bonferroni P**	**Slope**	**Bonferroni P**	**Slope**	**Bonferroni P**	**Slope**
*DROSHA*	9.42 × 10^−03^	-10.23	1.37 × 10^−05^	-22.12	5.19 × 10^−06^	-15.64
*DGCR8*	0.036	11.57	0.95	-0.46	0.37	6.23
*XPO5*	0.47	-3.03	1.38 × 10^−04^	-17.85	2.17 × 10^−03^	-10.74
*RAN*	0.27	0.49	0.14	-0.94	0.75	-0.12
*DICER1*	8.51 × 10^−03^	-13.77	1.97 × 10^−09^	-26.18	5.57 × 10^−10^	-21.72
*TRBP*	2.95 × 10^−05^	12.26	0.085	8.12	2.68 × 10^−04^	10.60
*EIF2C2*	0.022	-6.25	1.39 × 10^−07^	-9.07	1.88 × 10^−08^	-8.41

P-values and slopes from the linear regression of expression level of genes in the miRNA biogenesis pathway against mean RE-score, in the CEU, YRI and for both populations pooled.

**Figure 2:**
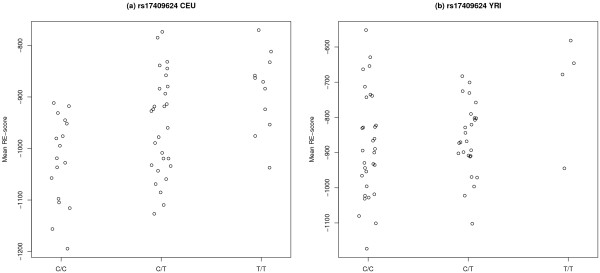
**Stripcharts for rs17409624.** Stripcharts of mean RE-score against genotype at rs17409624 in the (**a**) CEU and (**b**) YRI populations.

We carried out a second test of association, restricting to 336 SNPs that map to the genomic regions of these seven key genes involved in the miRNA biogenesis pathway. A SNP is mapped to the genomic region of a gene by dbSNP if it lies between 2kb upstream and 500bp downstream of the gene. Again there appear to be more low p-values than would be expected under the uniform distribution, pointing to a proportion of true positive associations in both populations (Additional file 2: Figure S3). The ten SNPs most strongly associated with mean RE-score in CEU and YRI are shown in Tables 3 and 4, respectively. One SNP, rs17409624, in an intron of *DROSHA* remained statistically significantly (*p*_*adjusted*_ < 0.05) associated with mean RE-score in the CEU following Bonferroni and permutation-based control for multiple testing. This SNP was also nominally significantly associated with mean RE-score in the YRI (*p* = 0.04); however, the minor allele frequency was much lower in YRI, limiting the power to detect an association with a significance that could survive multiple test correction. The magnitude and direction of the RE-score differences between genotypes are consistent across the two populations (Figure 2). Taken individually, the vast majority (214 of 244) of RE-scores are associated (*p* < 0.05) with genotype at this SNP in the CEU. This number drops to 36 of 244 in the YRI, however the lower minor allele frequency in YRI again limits the power to detect the association.

**Table 3:** Top 10 associations for miRNA biogenesis pathway related SNPs (CEU)

	**Location**	**Associated Gene**	**P-value**	**Bonferroni P**	**Q-value**	**Permutation**
						**p-value**
rs17409624	chr5:31,528,733	*DROSHA*	1.81 × 10^−04^	0.043	0.018	0.03
rs10078886	chr5:31,427,441	*DROSHA*	3.32 × 10^−04^	0.079	0.018	0.051
rs16901121	chr5:31,418,215	*DROSHA*	3.32 × 10^−04^	0.079	0.018	0.051
rs2279797	chr5:31,428,028	*DROSHA*	3.32 × 10^−04^	0.079	0.018	0.051
rs13183642	chr5:31,511,106	*DROSHA*	1.25 × 10^−03^	0.3	0.054	0.16
rs3805516	chr5:31,420,670	*DROSHA*	1.56 × 10^−03^	0.37	0.056	0.2
rs4867349	chr5:31,536,327	*DROSHA*	1.82 × 10^−03^	0.43	0.056	0.23
rs2287584	chr5:31,423,007	*DROSHA*	3.27 × 10^−03^	0.78	0.073	0.33
rs615344	chr5:31,425,788	*DROSHA*	3.39 × 10^−03^	0.8	0.073	0.34
rs682902	chr5:31,423,694	*DROSHA*	3.39 × 10^−03^	0.8	0.073	0.34

The results for association of miRNA biogenesis pathway related SNPs with mean RE-score in the CEU.

**Table 4:** Top 10 associations for miRNA pathway related SNPs (YRI)

	**Location**	**Associated Gene**	**P-value**	**Bonferroni P**	**Q-value**	**Permutation**
						**p-value**
rs6994531	chr8:141,544,476	*EIF2C2*	4.57 × 10^−03^	1	0.38	0.55
rs1633445	chr22:20,100,596	*DGCR8*	0.011	1	0.38	0.77
rs17409275	chr5:31,514,127	*DROSHA*	0.012	1	0.38	0.8
rs1209904	chr14:95,563,712	*DICER1*	0.015	1	0.38	0.86
rs1187650	chr14:95,551,554	*DICER1*	0.018	1	0.38	0.9
rs1187655	chr14:95,565,556	*DICER1*	0.018	1	0.38	0.9
rs6575499	chr14:95,622,200	*DICER1*	0.018	1	0.38	0.9
rs12881840	chr14:95,568,003	*DICER1*	0.020	1	0.38	0.9
rs12889800	chr14:95,618,898	*DICER1*	0.020	1	0.38	0.9
rs2292780	chr8:141,561,357	*EIF2C2*	0.022	1	0.38	0.93

As a further test of the association between rs17409624 and mean RE-score, we investigated the RE-scores of a particular class of intronic miRNAs (mirtrons), which are not processed by *DROSHA*[36]. If the association between the SNP and mean RE-score is real and is mediated by an effect on miRNA processing by *DROSHA*, the SNP should not be associated with the RE-scores of mirtrons. Consistent with this prediction, we found that a much lower proportion of mirtron RE-scores (based on TargetScan predictions from CEU RNA-seq data) are associated (at *α* = 0.05) with the *DROSHA* SNP (5 out of 12 mirtrons, compared to 214 out of 244 conventional miRNAs; *p* = 0.0004, from a two-sided Fisher’s exact test). We have found evidence that the subset of mirtrons that do show an association with the SNP do so because of an overlap between their target gene sets and the target gene sets of conventional miRNAs, as the mirtrons which are most significantly associated with rs17409624 tend to target genes that are also targeted by many other miRNAs; and mirtrons that target genes that are targeted by few conventional miRNAs are less significantly associated with rs17409624 (Additional file 2: Figure S4).

### Searching for causal SNPs

**Figure 3:**
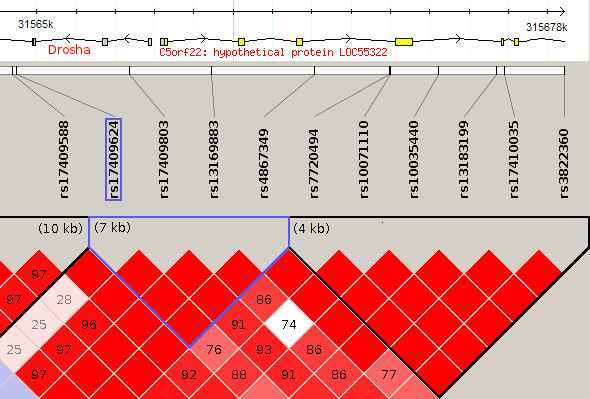
**Haplotypes in rs17409624 region.** Haplotype blocks around rs17409624 as calculated by confidence intervals in Haploview, using the HapMap CEU data. The block which includes rs17409624 is highlighted in blue; this block also includes the *DROSHA* promoter region.

We investigated the function of SNP rs17409624 using the “SNP Function Prediction” tool, which is part of the SNPinfo suite (available at http://www.niehs.nih.gov/snpinfo) [37]; however, no significant results were identified. We also searched the “GWAS Catalog” but did not find any previous studies which had identified this SNP [38]. To search for other SNPs that may be causally responsible for this association we used confidence intervals [39] as implemented in HaploView to calculate haplotype blocks for the CEU HapMap data. rs17409624 is located within a haplotype block that includes the *DROSHA* promoter region (Figure 3). We verified that this is the active promoter of *DROSHA* using data recently released by the ENCODE project *et al.*[40]. Chromatin states for this locus are shown in Additional file 2: Figure S5. The expression level of *DROSHA* is significantly associated with mean RE-score (Table 2); however, the genotype of this locus was not significantly correlated with *DROSHA* expression level (p = 0.39) or with the relative expression level of any *DROSHA* transcript isoforms (identified using Cufflinks [41]). A further possibility is that rs17409624 is in linkage disequilibrium (LD) with an exonic SNP that was not genotyped on the HapMap microarrays. Using SNP calls from genome sequence data released by the 1,000 Genomes Project [42] we found no coding SNPs with a stronger association to mean RE-score than rs17409624, the regions assayed included the 3′ and 5′ UTRs. We caution however, that there was much less statistical power to detect an association using the 1,000 Genomes data, as there was an overlap of only 45 samples between the 1,000 Genomes Project dataset (versus 59 for the HapMap microarray data) and the RNA-seq samples from the CEU used in this analysis, which means that it is difficult to rule out the possibility of linkage of rs17409624 with a causative SNP in the coding region. These results are provided in Additional file 4. Thus, the causal mechanism linking genetic variation at the *DROSHA* locus to variation in the RE-score remains unclear.

### Integrative analysis of miRNA expression and RE-score data

miRNA expression data has recently been generated for some of the HapMap CEU and YRI cell lines [43]. In the majority of cases, miRNA expression levels and their corresponding RE-scores were not significantly correlated. Average Spearman correlation between miRNA expression and corresponding TargetScan based RE-score from the RNA-seq data is only 0.009 in the CEU and -0.0003 in the YRI. Although surprising, this observation is consistent with the findings of Cheng *et al*. [13], who, for the original RE-score study, performed Spearman correlations of the t-scores of comparisons of miRNA expression and RE-scores between ER- and ER+ breast cancers, finding only very weak positive correlation. Similar results have also been observed on two separate datasets by Liang *et al.*[44]. Correlations between miRNA expression level and RE-scores are included in Additional file 5. However, we find that in the CEU, the expression of 17 of 201 miRNAs that were consistently expressed across the cell lines is associated (*p*<0.05) with rs17409624 and that 13 of these associations are in the same direction as mean RE-score. One miRNA is associated with the SNP in the YRI, but once again, the lower minor allele frequency of rs17409624 in the YRI limits the power to identify associations. P-values and false discovery rates for these 18 miRNAs (17 CEU and 1 YRI) for genotype association are included in Additional file 6. Thus, this SNP represents a trans-eQTL cluster for miRNA gene expression. We hypothesize that this trans-eQTL reflects inter-individual differences in the efficiency of miRNA processing by *DROSHA*. Given that miRNA expression measurements are relative (in this case miRNA expression was measured using a pooled reference microarray design), it is possible that this polymorphism may affect the absolute copy numbers of a large fraction of miRNAs, even though an association between miRNA expression and the SNP is detectable for a relatively small fraction of miRNAs. This hypothesis could be tested using transcriptome sequencing strategies designed to measure the abundance of miRNAs relative to other RNA species. Indeed, given a global and consistent change in expression of all miRNAs in a sample, one may not expect the expression of any miRNAs to be associated with rs17409624, as the proportion of the transcript pool occupied by any given miRNA, would remain unchanged. However, the miRNA regulatory effect polymorphism need not affect the expression of all miRNAs to exactly the same degree, potentially leading to both positive and negative associations of miRNA expression with the SNP.

As discussed above, RE-scores of the majority of miRNAs were not correlated with miRNA expression. This remained the case when we restricted to miRNAs whose expression varied most across samples. However, the RE-scores of individual miRNAs were correlated with the mean RE-score calculated across all miRNAs. We restricted this analysis to the 20 most variable miRNAs. Of the top 20 in either population, 14 in the CEU and 13 in the YRI had TargetScan prediction data and therefore RE-scores. We only considered these highly variable miRNAs because quantities that are relatively constant across samples are not expected to be correlated, given the noise inherent in microarray data. The correlation between mean and individual miRNA RE-scores is not simply a consequence of overlaps in genes targeted by different miRNAs, since it holds true even when the mean RE-score is recalculated, for each miRNA correlation test, after all of the individual miRNAs targets have been subtracted from the target sets of the remaining miRNAs. 13 of the 14 highly varying miRNAs in the CEU and all 13 of 13 in the YRI show a stronger association between the individual RE-score and (subtracted) mean RE-score, than between the individual RE-score and the expression of the miRNA itself. In most cases this difference is large (Additional file 7), hence, the mean RE-score in a sample may be a much better predictor of the expression level of the targets of any particular miRNA, than is the expression profile of the miRNA itself. It is, perhaps, not surprising that the expression level of an individual miRNA is not indicative of the expression of its target genes, given that targeted genes are often targets of a large number of miRNAs. Of 11,759 genes which are predicted to be targeted by at least one miRNA (by the full TargetScan set), the average number of miRNAs targeting each gene is 17.48. In this context, the fact that the mean RE-score has power to predict the expression levels of a miRNA target, even when the mean RE-score is calculated without considering the targets of that miRNA is interesting and points to differences in the effect of the miRNA pathway on target genes across the cell lines.

## Conclusions

We have found evidence of heritability of the regulatory effect of miRNAs in human. We have also identified an association between the regulatory effect of miRNAs and a SNP in the miRNA processing gene *DROSHA*. This association was identified in lymphoblastoid cell lines and it remains to be seen whether and in which primary cells the regulatory effect of miRNAs is associated with the *DROSHA* locus. As noted in the Background, Cheng *et al.* had observed that there is a change in miRNA RE-scores between ER- and ER+ breast cancer subtypes. Thomsom *et al.* showed that mature miRNA levels are generally lower in several human primary cancers, despite unchanged pri-miRNA levels and this has been attributed to defective processing by *DROSHA*[45], while *DROSHA* and *DICER* have also been shown to be downregulated in endometrial cancer and specific subgroups of breast cancer [46,47]. Thus, it will be important to investigate further the phenotypic consequences of inter-individual differences in miRNA regulatory efficiency and the influence on gene expression, possible tumorigenesis and the impact of such inter-individual differences in the context of the use of miRNAs as biomarkers.

## Methods

### Data

Raw gene expression microarray data of related individuals from the CEU and YRI populations of the HapMap project were downloaded from GEO under accession number *GSE7792*, these data were generated by Huang *et al.*[23] using Affymetrix Human Exon 1.0 ST microarrays. Prior to calculating gene expression level estimates, the data were RMA normalized [48] and genes whose expression level were below the detection threshold, as estimated by the DABG algorithm (*p* < 0.05), were set to zero; these steps were performed using Affymetrix Power Tools and R as described in [49]. RNA-seq data for unrelated individuals of the HapMap YRI population were generated by Pickrell *et. al*[21] and we obtained these aligned data from GEO under accession number *GSE19480*. Similarly, Montgomery *et al.*[22] used RNA-seq to assess gene expression of unrelated CEU samples and these data were obtained from ArrayExpress under accession number *E-MTAB-197*. All data were aligned to *hg18* using MAQ [50]. We performed gene expression analysis using R/Bioconductor. Data were loaded in R [51] using the *ShortRead*[52] library. Following Montgomery *et al.*, only reads that had a mapping quality score of greater than or equal to 10 were included. The *GenomicRanges*[53] library was used to compute the number of reads mapping to exons of each gene and expression values were normalized using the RPKM [54] procedure. miRNA prediction data were obtained using the R library *RmiR.Hs.miRNA*[55] which provides a database of miRNA targets for several widely used algorithms. The HapMap release 28 (merged data for phases I, II and III) [19,20] SNP data were downloaded from the HapMap website, converted to GenABEL format and trimmed to include only samples in the CEU and YRI populations for which there was matching RNA-seq data.

### Estimating Heritability of mean RE-score

Narrow sense heritability of individual miRNA RE-scores and mean RE-score was estimated using a robust linear regression model [24,25]. The *rlm()* function from the R library *MASS* was used to fit regression models for child value dependent on mean of parents. Population of origin was included as a factor in the models. The slope of the regression line provides an estimate of heritability.

### Permutation testing of heritability of mean RE-score

To calculate a corrected p-value for heritability of mean RE-score of a miRNA prediction algorithm, we performed 1,000 permutations of the prediction algorithm’s miRNA gene target sets and recalculated heritability of mean RE-score following each permutation; the permutation p-value was the proportion of permuted sets that return p-values which are equal to, or lower than, the original raw p-value for that algorithm. To perform a permutation, we replace each gene target of each miRNA’s target set with a randomly chosen gene, but only genes for which expression data is available are replaced or used for replacement, as only these can affect RE-scores. If a gene is a target of multiple microRNAs, it is replaced by the same randomly chosen gene in every target set, so as to maintain the structure of the data.

### Genome-wide association test

The R package *GenABEL*[56,57] was used for filtering and tests of association. Prior to testing for association, genotype data were filtered as follows. Obvious close relatives are removed by discarding the child samples and to avoid the effects of population stratification CEU and YRI samples are assayed separately. Markers with a low minor allele frequency were filtered by excluding SNPs for which there were less than 5 copies of the minor allele across all samples. We used only SNPs genotyped as part of HapMap phase III. Individuals or SNPs were excluded for a call rate of < 0.95. Tests for Hardy-Weinberg equilibrium were conducted using Pearson’s *χ*^2^, comparing observed genotype frequencies in the data to the calculated expected frequencies; a cut-off FDR level of 0.2 was applied. To assess if any remaining relatedness exists among the samples, the pairwise proportion of alleles identical-by-state (IBS) was calculated between all individuals based on 2,000 randomly chosen autosomal markers, ensuring IBS < 0.95 for all samples. For multiple testing correction of association p-values, permutations were calculated by permuting phenotype labels and performing tests of association as normal; for each raw p-value, we computed the number of permutations for which a p-value equal to, or lower than, the original raw p-value was reached and divide this by the number of permutations, the result of which is the adjusted p-value. False discovery rates were also assessed using the R package *qvalue*[58].

### Calculating association between individual miRNA RE-score, mean RE-score and miRNA expression

For each of 14 highly varying miRNAs in the CEU samples and 13 in the YRI, we fit a multiple linear regression model of individual miRNA RE-score dependent on the expression of the miRNA and the mean RE-score. For each fit of the model, mean RE-score was re-calculated with the genes that are targets of the particular individual miRNA removed from the gene expression matrix, so as to avoid a bias in the association between the two variables.

## Competing interests

The authors declare that they have no competing interests.

## Author’s contributions

PG performed the analysis and drafted the manuscript. CS conceived the study. CS and AG directed the project. RSH and ERG generated the miRNA expression data and provided guidance on analysis. All authors read and approved the final manuscript.

## Supplementary Material

Additional file 1 Tables of regression p-values and slopes, for heritability of individual miRNA RE-scores from TargetScan and PicTar algorithms. Click here for file

Additional file 2 Figure S1. Heritability for individual RE-scores. Histograms of p-values for tests of heritability of individual RE-scores for (a) TargetScan and (b) PicTar algorithms. Figure S2: P-Values for genome-wide tests of association. Histograms (a & b) of p-values for tests of association between all SNP markers and mean RE-score and Manhattan plots (c & d) of p-values in the CEU and YRI respectively. Figure S3: Histograms of p-values for miRNA biogenesis pathway SNPs. Histograms of p-values for the tests of association between SNP markers mapped to the miRNA biogenesis pathway and mean RE-score in the (a) CEU and (b) YRI populations. Figure S4: Many mirtron target genes are also miRNA targets Relationship between the strength of association with rs17409624 for mirtrons and the average number of conventional miRNAs that also target the mirtron’s target genes. This figure is based on TargetScan predictions for conserved miRNA families on HapMap CEU data. *R*^2^ = 0.65, *p* = 5.1 × 10^−4^ Figure S5: *DROSHA* promoter region Chromatin state of *DROSHA* region for nine cell lines from the ENCODE project. Active promoter is shown in bright red. The haplotype block for rs17409624 is shown in black and clearly overlaps the promoter region. Click here for file

Additional file 3 Tables of top 10 SNPs from the genome-wide screen for association of mean RE-score and SNP genotypes, in the CEU and in the YRI. Click here for file

Additional file 4 Table of p-values for association of Drosha coding SNPs and mean RE-score, based on the 1,000 genomes CEU data. Only SNPs with more than 1 copy of the minor allele in the 45 samples available are included. If a SNP was genotyped by the HapMap project, that p-value is also included. Click here for file

Additional file 5 Tables of Spearman correlations and p-values for correlations between individual miRNAs and their associated RE-scores in the CEU and YRI. Click here for file

Additional file 6 Tables of P-values and False discovery rates (calculated using the *qvalue* package in R) for association between the expression of individual miRNAs and rs17409624. MiRNAs with *p* < 0.05 are shown. Click here for file

Additional file 7 P-values from linear models of individual RE-score dependent on (subtracted) mean RE-score and miRNA expression. Click here for file

## References

[B1] Krol J Loedige I Filipowicz W The widespread regulation of microRNA biogenesis, function and decay Nat Rev Genet 2010 11 9 597 610 [http://dx.doi.org/10.1038/nrg2843] 20661255 10.1038/nrg2843

[B2] Grimson A Farh KKH Johnston WK Garrett-Engele P Lim LP Bartel DP MicroRNA targeting specificity in mammals: determinants beyond seed pairing Mol Cell 2007 27 91 105 [http://dx.doi.org/10.1016/j.molcel.2007.06.017] 10.1016/j.molcel.2007.06.017 17612493 PMC3800283

[B3] Chekulaeva M Filipowicz W Mechanisms of miRNA-mediated post-transcriptional regulation in animal cells Curr Opin Cell Biol 2009 21 3 452 460 [http://dx.doi.org/10.1016/j.ceb.2009.04.009] 10.1016/j.ceb.2009.04.009 19450959

[B4] Bartel DP MicroRNAs: target recognition and regulatory functions Cell 2009 136 2 215 233 [http://dx.doi.org/10.1016/j.cell.2009.01.002] 10.1016/j.cell.2009.01.002 19167326 PMC3794896

[B5] Xu P Guo M Hay BA MicroRNAs and the regulation of cell death Trends Genet 2004 20 12 617 624 [http://dx.doi.org/10.1016/j.tig.2004.09.010] 10.1016/j.tig.2004.09.010 15522457

[B6] Cheng AM Byrom MW Shelton J Ford LP Antisense inhibition of human miRNAs and indications for an involvement of miRNA in cell growth and apoptosis Nucleic Acids Res 2005 33 4 1290 1297 [http://dx.doi.org/10.1093/nar/gki200] 10.1093/nar/gki200 15741182 PMC552951

[B7] Visone R Croce CM MiRNAs and cancer Am J Pathol 2009 174 4 1131 1138 [http://dx.doi.org/10.2353/ajpath.2009.080794] 10.2353/ajpath.2009.080794 19264914 PMC2671346

[B8] Eis PS Tam W Sun L Chadburn A Li Z Gomez MF Lund E Dahlberg JE Accumulation of miR-155 and BIC RNA in human B cell lymphomas Proc Natl Acad Sci U S A 2005 102 10 3627 3632 [http://dx.doi.org/10.1073/pnas.0500613102] 10.1073/pnas.0500613102 15738415 PMC552785

[B9] Cimmino A Calin GA Fabbri M Iorio MV Ferracin M Shimizu M Wojcik SE Aqeilan RI Zupo S Dono M Rassenti L Alder H Volinia S Liu CG Kipps TJ Negrini M Croce CM miR-15 and miR-16 induce apoptosis by targeting BCL2 Proc Natl Acad Sci U S A 2005 102 39 13944 13949 [http://dx.doi.org/10.1073/pnas.0506654102] 10.1073/pnas.0506654102 16166262 PMC1236577

[B10] Calin GA Croce CM MicroRNAs and chromosomal abnormalities in cancer cells Oncogene 2006 25 46 6202 6210 [http://dx.doi.org/10.1038/sj.onc.1209910] 10.1038/sj.onc.1209910 17028600

[B11] Winter J Jung S Keller S Gregory RI Diederichs S Many roads to maturity: microRNA biogenesis pathways and their regulation Nat Cell Biol 2009 11 3 228 234 [http://dx.doi.org/10.1038/ncb0309-228] 10.1038/ncb0309-228 19255566

[B12] Guo H Ingolia NT Weissman JS Bartel DP Mammalian microRNAs predominantly act to decrease target mRNA levels Nature 2010 466 835 840 10.1038/nature09267 20703300 PMC2990499

[B13] Cheng C Fu X Alves P Gerstein M mRNA expression profiles show differential regulatory effects of microRNAs between estrogen receptor-positive and estrogen receptor-negative breast cancer Genome Biol 2009 10 9 R90 [http://dx.doi.org/10.1186/gb-2009-10-9-r90] 10.1186/gb-2009-10-9-r90 19723326 PMC2768979

[B14] Yu Z Jian Z Shen SH Purisima E Wang E Global analysis of microRNA target gene expression reveals that miRNA targets are lower expressed in mature mouse and Drosophila tissues than in the embryos Nucleic Acids Res 2007 35 152 164 [http://dx.doi.org/10.1093/nar/gkl1032] 17158157 10.1093/nar/gkl1032 PMC1802562

[B15] Cheng C Li LM Inferring microRNA activities by combining gene expression with microRNA target prediction PLoS One 2008 3 4 e1989 [http://dx.doi.org/10.1371/journal.pone.0001989] 10.1371/journal.pone.0001989 18431476 PMC2291556

[B16] Arora A Simpson DA Individual mRNA expression profiles reveal the effects of specific microRNAs Genome Biol 2008 9 5 R82 [http://dx.doi.org/10.1186/gb-2008-9-5-r82] 10.1186/gb-2008-9-5-r82 18485210 PMC2441468

[B17] Wang X Wang X Systematic identification of microRNA functions by combining target prediction and expression profiling Nucleic Acids Res 2006 34 5 1646 1652 [http://dx.doi.org/10.1093/nar/gkl068] 10.1093/nar/gkl068 16549876 PMC1405822

[B18] Buffa FM Camps C Winchester L Snell CE Gee HE Sheldon H Taylor M Harris AL Ragoussis J microRNA associated progression pathways and potential therapeutic targets identified by integrated mRNA and microRNA expression profiling in breast cancer Cancer Res 2011 [http://dx.doi.org/10.1158/0008-5472.CAN-11-0489] 10.1158/0008-5472.CAN-11-0489 21737487

[B19] Consortium IH The International HapMap Project Nature 2003 426 6968 789 796 10.1038/nature02168 14685227

[B20] International HapMap Consortium A second generation human haplotype map of over 3.1 million SNPs Nature 2007 449 7164 851 861 [http://dx.doi.org/10.1038/nature06258] 10.1038/nature06258 17943122 PMC2689609

[B21] Pickrell JK Marioni JC Pai AA Degner JF Engelhardt BE Nkadori E Veyrieras JB Stephens M Gilad Y Pritchard JK Understanding mechanisms underlying human gene expression variation with RNA sequencing Nature 2010 464 7289 768 772 [http://dx.doi.org/10.1038/nature08872] 10.1038/nature08872 20220758 PMC3089435

[B22] Montgomery SB Sammeth M Gutierrez-Arcelus M Lach RP Ingle C Nisbett J Guigo R Dermitzakis ET Transcriptome genetics using second generation sequencing in a Caucasian population Nature 2010 464 7289 773 777 [http://dx.doi.org/10.1038/nature08903] 10.1038/nature08903 20220756 PMC3836232

[B23] Huang RS Duan S Bleibel WK Kistner EO Zhang W Clark TA Chen TX Schweitzer AC Blume JE Cox NJ Dolan ME A genome-wide approach to identify genetic variants that contribute to etoposide-induced cytotoxicity Proc Natl Acad Sci U S A 2007 104 23 9758 9763 [http://dx.doi.org/10.1073/pnas.0703736104] 10.1073/pnas.0703736104 17537913 PMC1887589

[B24] Visscher PM Hill WG Wray NR Heritability in the genomics era–concepts and misconceptions Nat Rev Genet 2008 9 4 255 266 [http://dx.doi.org/10.1038/nrg2322] 18319743 10.1038/nrg2322

[B25] Wray PN Visscher Estimating Trait Heritability Nature Education 2008

[B26] Hamilton MB Population Genetics 2009 John Wiley & Sons

[B27] Lewis BP Burge CB Bartel DP Conserved seed pairing, often flanked by adenosines, indicates that thousands of human genes are microRNA targets Cell 2005 120 15 20 [http://dx.doi.org/10.1016/j.cell.2004.12.035] 10.1016/j.cell.2004.12.035 15652477

[B28] Krek A Grün D Poy MN Wolf R Rosenberg L Epstein EJ MacMenamin P da Piedade I Gunsalus KC Stoffel M Rajewsky N Combinatorial microRNA target predictions Nat Genet 2005 37 5 495 500 [http://dx.doi.org/10.1038/ng1536] 10.1038/ng1536 15806104

[B29] Baek D Villén J Shin C Camargo FD Gygi SP Bartel DP The impact of microRNAs on protein output Nature 2008 455 7209 64 71 [http://dx.doi.org/10.1038/nature07242] 10.1038/nature07242 18668037 PMC2745094

[B30] John B Enright AJ Aravin A Tuschl T Sander C Marks DS Human MicroRNA targets PLoS Biol 2004 2 11 e363 [http://dx.doi.org/10.1371/journal.pbio.0020363] 10.1371/journal.pbio.0020363 15502875 PMC521178

[B31] Wang X Naqa IME Prediction of both conserved and nonconserved microRNA targets in animals Bioinformatics 2008 24 3 325 332 [http://dx.doi.org/10.1093/bioinformatics/btm595] 10.1093/bioinformatics/btm595 18048393

[B32] Griffiths-Jones S Saini HK van Dongen S Enright AJ miRBase: tools for microRNA genomics Nucleic Acids Res 2008 36 Database issue D154 D158 [http://dx.doi.org/10.1093/nar/gkm952] 17991681 10.1093/nar/gkm952 PMC2238936

[B33] Cheung VG Conlin LK Weber TM Arcaro M Jen KY Morley M Spielman RS Natural variation in human gene expression assessed in lymphoblastoid cells Nat Genet 2003 33 3 422 425 [http://dx.doi.org/10.1038/ng1094] 10.1038/ng1094 12567189

[B34] Schadt EE Monks SA Drake TA Lusis AJ Che N Colinayo V Ruff TG Milligan SB Lamb JR Cavet G Linsley PS Mao M Stoughton RB Friend SH Genetics of gene expression surveyed in maize, mouse and man Nature 2003 422 6929 297 302 [http://dx.doi.org/10.1038/nature01434] 10.1038/nature01434 12646919

[B35] Brem RB Yvert G Clinton R Kruglyak L Genetic dissection of transcriptional regulation in budding yeast Science 2002 296 5568 752 755 [http://dx.doi.org/10.1126/science.1069516] 10.1126/science.1069516 11923494

[B36] Berezikov E Chung WJ Willis J Cuppen E Lai EC Mammalian mirtron genes Mol Cell 2007 28 2 328 336 [http://dx.doi.org/10.1016/j.molcel.2007.09.028] 10.1016/j.molcel.2007.09.028 17964270 PMC2763384

[B37] Xu Z Taylor JA SNPinfo: integrating GWAS and candidate gene information into functional SNP selection for genetic association studies Nucleic Acids Res 2009 37 Web Server issue W600 W605 [http://dx.doi.org/10.1093/nar/gkp290] 19417063 10.1093/nar/gkp290 PMC2703930

[B38] Hindorff LA Sethupathy P Junkins HA Ramos EM Mehta JP Collins FS Manolio TA Potential etiologic and functional implications of genome-wide association loci for human diseases and traits Proc Natl Acad Sci U S A 2009 106 23 9362 9367 [http://dx.doi.org/10.1073/pnas.0903103106] 10.1073/pnas.0903103106 19474294 PMC2687147

[B39] Gabriel SB Schaffner SF Nguyen H Moore JM Roy J Blumenstiel B Higgins J DeFelice M Lochner A Faggart M Liu-Cordero SN Rotimi C Adeyemo A Cooper R Ward R Lander ES Daly MJ Altshuler D The structure of haplotype blocks in the human genome Science 2002 296 5576 2225 2229 [http://dx.doi.org/10.1126/science.1069424] 10.1126/science.1069424 12029063

[B40] Ernst J Kheradpour P Mikkelsen TS Shoresh N Ward LD Epstein CB Zhang X Wang L Issner R Coyne M Ku M Durham T Kellis M Bernstein BE Mapping and analysis of chromatin state dynamics in nine human cell types Nature 2011 473 7345 43 49 [http://dx.doi.org/10.1038/nature09906] 10.1038/nature09906 21441907 PMC3088773

[B41] Trapnell C Williams BA Pertea G Mortazavi A Kwan G van Baren MJ Salzberg SL Wold BJ Pachter L Transcript assembly and quantification by RNA-Seq reveals unannotated transcripts and isoform switching during cell differentiation Nat Biotechnol 2010 28 5 511 515 [http://dx.doi.org/10.1038/nbt.1621] 10.1038/nbt.1621 20436464 PMC3146043

[B42] Consortium GP A map of human genome variation from population-scale sequencing Nature 2010 467 7319 1061 1073 [http://dx.doi.org/10.1038/nature09534] 10.1038/nature09534 20981092 PMC3042601

[B43] Huang RS Gamazon ER Ziliak D Wen Y Im HK Zhang W Wing C Duan S Bleibel WK Cox NJ Dolan ME Population differences in microRNA expression and biological implications RNA Biol 2011 8 4 692 701 [http://dx.doi.org/10.4161/rna.8.4.16029] 10.4161/rna.8.4.16029 21691150 PMC3225983

[B44] Liang Z Zhou H Zheng H Wu J Expression levels of microRNAs are not associated with their regulatory activities Biol Direct 2011 6 43 [http://dx.doi.org/10.1186/1745-6150-6-43] 10.1186/1745-6150-6-43 21929766 PMC3189187

[B45] Thomson JM Newman M Parker JS Morin-Kensicki EM Wright T Hammond SM Extensive post-transcriptional regulation of microRNAs and its implications for cancer Genes Dev 2006 20 16 2202 2207 [http://dx.doi.org/10.1101/gad.1444406] 10.1101/gad.1444406 16882971 PMC1553203

[B46] Torres A Torres K Paszkowski T Jodlowska-Jedrych B Radomanski T Ksiazek A Maciejewski R Major regulators of microRNAs biogenesis Dicer and Drosha are down-regulated in endometrial cancer Tumour Biol 2011 32 4 769 776 [http://dx.doi.org/10.1007/s13277-011-0179-0] 10.1007/s13277-011-0179-0 21559780 PMC3131523

[B47] Dedes KJ Natrajan R Lambros MB Geyer FC Lopez-Garcia MA Savage K Jones RL Reis-Filho JS Down-regulation of the miRNA master regulators Drosha and Dicer is associated with specific subgroups of breast cancer Eur J Cancer 2011 47 138 150 [http://dx.doi.org/10.1016/j.ejca.2010.08.007] 10.1016/j.ejca.2010.08.007 20832293

[B48] Irizarry RA Hobbs B Collin F Beazer-Barclay YD Antonellis KJ Scherf U Speed TP Exploration, normalization, and summaries of high density oligonucleotide array probe level data Biostatistics 2003 4 2 249 264 [http://dx.doi.org/10.1093/biostatistics/4.2.249] 10.1093/biostatistics/4.2.249 12925520

[B49] Lockstone HE Exon array data analysis using Affymetrix power tools and R statistical software Brief Bioinform 2011 12 6 634 644 [http://dx.doi.org/10.1093/bib/bbq086] 10.1093/bib/bbq086 21498550 PMC3220870

[B50] Li H Ruan J Durbin R Mapping short DNA sequencing reads and calling variants using mapping quality scores Genome Res 2008 18 11 1851 1858 [http://dx.doi.org/10.1101/gr.078212.108] 10.1101/gr.078212.108 18714091 PMC2577856

[B51] R Development Core Team R: A Language and Environment for Statistical Computing 2010 R Foundation for Statistical Computing, Vienna, Austria [http://www.R-project.org. [ISBN 3-900051-07-0]

[B52] Morgan M Lawrence M Anders S ShortRead: Base classes and methods for high-throughput short-read sequencing data [R package version 1.6.2]

[B53] Aboyoun P Pages H Lawrence M GenomicRanges: Representation and manipulation of genomic intervals [R package version 1.0.1]

[B54] Mortazavi A Williams BA McCue K Schaeffer L Wold B Mapping and quantifying mammalian transcriptomes by RNA-Seq Nat Methods 2008 5 7 621 628 [http://dx.doi.org/10.1038/nmeth.1226] 10.1038/nmeth.1226 18516045 PMC13303166

[B55] Favero F RmiR.Hs.miRNA: Various databases of microRNA Targets [R package version 1.0.6]

[B56] Aulchenko YS Ripke S Isaacs A van Duijn CM GenABEL: an R library for genome-wide association analysis Bioinformatics 2007 23 1294 1296 10.1093/bioinformatics/btm108 17384015

[B57] Aulchenko YS Struchalin MV van Duijn CM ProbABEL package for genome-wide association analysis of imputed data BMC Bioinformatics 2010 11 134 10.1186/1471-2105-11-134 20233392 PMC2846909

[B58] Storey JD Tibshirani R Statistical significance for genomewide studies Proc Natl Acad Sci U S A 2003 100 16 9440 9445 [http://dx.doi.org/10.1073/pnas.1530509100] 10.1073/pnas.1530509100 12883005 PMC170937

