# Supplementary material for: The regulatory effect of miRNAs is a heritable genetic trait in humans
Source: BMC Genomics. 2012 Aug 10;13:383. doi: 10.1186/1471-2164-13-383 (PMC3532363; doi:10.1186/1471-2164-13-383)
Supplement: Additional file 2 — Figure S1. Heritability for individual RE-scores. Histograms of p-values for tests of heritability of individual RE-scores for (a) TargetScan and (b) PicTar algorithms. Figure S2: P-Values for genome-wide tests of association. Histograms (a & b) of p-values for tests of association between all SNP markers and mean RE-score and Manhattan plots (c & d) of p-values in the CEU and YRI respectively. Figure S3: Histograms of p-values for miRNA biogenesis pathway SNPs. Histograms of p-values for the tests of association between SNP markers mapped to the miRNA biogenesis pathway and mean RE-score in the (a) CEU and (b) YRI populations. Figure S4: Many mirtron target genes are also miRNA targets Relationship between the strength of association with rs17409624 for mirtrons and the average number of conventional miRNAs that also target the mirtron’s target genes. This figure is based on TargetScan predictions for conserved miRNA families on HapMap CEU data. R2 = 0.65, p = 5.1 × 10^−4^ Figure S5: DROSHA promoter region Chromatin state of DROSHA region for nine cell lines from the ENCODE project. Active promoter is shown in bright red. The haplotype block for rs17409624 is shown in black and clearly overlaps the promoter region. [file 1471-2164-13-383-S2.zip › FigureS5.pdf]

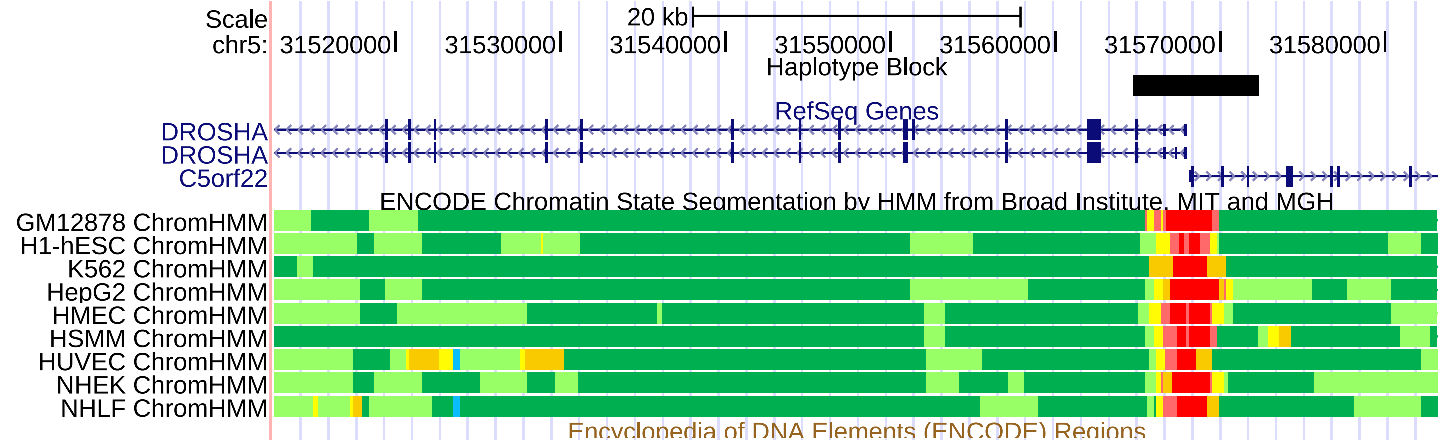
Scale
20 kb
chr5:
31520000
31530000
31540000
31550000
31560000
31570000
31580000
Haplotype Block
RefSeq Genes
DROSHA
DROSHA
C5orf22
ENCODE Chromatin State Segmentation by HMM from Broad Institute, MIT and MGH
GM12878 ChromHMM
H1-hESC ChromHMM
K562 ChromHMM
HepG2 ChromHMM
HMEC ChromHMM
HSMM ChromHMM
HUVEC ChromHMM
NHEK ChromHMM
NHLF ChromHMM
Encyclopedia of DNA Elements (ENCODE) Regions
