# Supplementary material for: The regulatory effect of miRNAs is a heritable genetic trait in humans
Source: BMC Genomics. 2012 Aug 10;13:383. doi: 10.1186/1471-2164-13-383 (PMC3532363; doi:10.1186/1471-2164-13-383)
Supplement: Additional file 3 — Tables of top 10 SNPs from the genome-wide screen for association of mean RE-score and SNP genotypes, in the CEU and in the YRI. [file 1471-2164-13-383-S3.zip › AdditionalFile3_ tables of top values from genome wide screen/AdditionalFile3_GWAS_results.pdf]

Supplementary Table 1 - GWAS Results (CEU)						
**SNP**	**Location**	**Associated Gene**	**P-Val.**	**Bonf. P-Val.**	**qValue**	**Perm P-Val.**
rs10145516	14:37,791,746	CLEC14A	1.36×10^{-07}	0.15	0.075	0.69
rs10145633	14:37,792,029	CLEC14A	1.36×10^{-07}	0.15	0.075	0.69
rs429166	20:44,631,678	SLC13A3	6.26×10^{-07}	0.69	0.17	0.96
rs436978	20:44,631,938	SLC13A3	6.26×10^{-07}	0.69	0.17	0.96
rs7783926	7:54,773,333	None	1.05×10^{-06}	1	0.18	1
rs11060151	12:128,181,280	TMEM132D	1.20×10^{-06}	1	0.18	1
rs12339206	9:115,472,577	ENSG00000227482	1.64×10^{-06}	1	0.18	1
rs1950351	14:93,526,800	None	1.66×10^{-06}	1	0.18	1
rs171101	5:135,646,103	TRPC7	2.23×10^{-06}	1	0.18	1
rs1392170	5:135,659,201	TRPC7	2.23×10^{-06}	1	0.18	1

Supplementary Table 2 - GWAS Results (YRI)						
**SNP**	**Location**	**Associated Gene**	**P-Val.**	**Bonf. P-Val.**	**qValue**	**Perm P-Val.**
rs35176	16:63,607,893	CDH11	4.50×10^{-08}	0.054	0.035	0.49
rs2280396	16:63,630,040	CDH11	5.84×10^{-08}	0.07	0.035	0.56
rs6559987	9:89,235,621	None	1.73×10^{-07}	0.21	0.069	0.82
rs2498766	23:133,947,781	Z83826.1	4.26×10^{-07}	0.51	0.11	0.96
rs6501775	17:70,822,168	ENSG00000257073	5.38×10^{-07}	0.64	0.11	0.98
rs4241991	4:55,626,003	ENSG00000250646	6.72×10^{-07}	0.8	0.11	0.99
rs16827088	1:40,486,621	TMCO2	7.81×10^{-07}	0.93	0.11	1
rs4738541	8:57,803,283	None	8.16×10^{-07}	0.98	0.11	1
rs10949013	6:57,237,646	None	8.32×10^{-07}	1	0.11	1
rs7337798	13:100,944,173	ITGBL1	1.01×10^{-06}	1	0.12	1
